# Supplementary material for: Plasticity of gene expression according to salinity in the testis of broodstock and F1 black-chinned tilapia, Sarotherodon melanotheron heudelotii
Source: PeerJ. 2014 Dec 18;2:e702. doi: 10.7717/peerj.702 (PMC4273931; doi:10.7717/peerj.702)
Supplement: Table S1 [file peerj-02-702-s001.docx]

Supplementary Table 1. Annotation features of the 23 non-responsive genes

| Sequence name^a^ | Sequence length (nt) | #BlastX hits^b^ | Protein description^c^ | E-Value | #GO terms |
| --- | --- | --- | --- | --- | --- |
| Transcript_AVA1_35277 | 482 | 20 | PREDICTED: type-2 ice-structuring protein-like isoform X1 | 1.12809E-78 | 0 |
| Contig_Tilapia_90_42 | 1200 | 20 | PREDICTED: steroidogenic acute regulatory protein, mitochondrial-like | 0.0 | 4 |
| Transcript_AVA2_28399 | 215 | 20 | PREDICTED: spermatogenesis-associated protein 6-like isoform X1 | 6.14314E-33 | 3 |
| Contig_Tilapia_90_2464 | 318 | 20 | PREDICTED: serine/threonine-protein kinase MRCK beta-like | 7.65537E-63 | 11 |
| Transcript_AVA3_18623 | 241 | 17 | PREDICTED: S100P-binding protein-like isoform X1 | 1.67897E-39 | 2 |
| Transcript_AVA1_55478 | 318 | 20 | PREDICTED: ropporin-1-like protein-like isoform X1 | 4.89654E-64 | 4 |
| Transcript_AVA3_14200 | 287 | 20 | PREDICTED: proteasome subunit beta type-4-like | 9.87423E-36 | 5 |
| Contig_Tilapia_90_2469 | 364 | 20 | ND5 gene product (mitochondrion) | 3.78555E-63 | 8 |
| Transcript_AVA1_4937 | 412 | 20 | PREDICTED: NADH dehydrogenase 1 alpha subcomplex subunit 4-like | 5.47508E-52 | 8 |
| Contig_Tilapia_90_10837 | 594 | 20 | PREDICTED: homologous-pairing protein 2 homolog isoform X1 | 1.27384E-137 | 0 |
| Contig_Tilapia_90_14414 | 151 | 20 | PREDICTED: high mobility group protein B2-like | 9.3765E-26 | 2 |
| Transcript_AVA1_52992 | 141 | 20 | PREDICTED: GTP-binding nuclear protein Ran-like, partial | 4.03386E-27 | 28 |
| Transcript_AVA2_4300 | 283 | 20 | PREDICTED: glyceraldehyde-3-phosphate dehydrogenase-like isoform X1 | 2.13313E-25 | 5 |
| Transcript_AVA1_9958 | 312 | 20 | PREDICTED: dynein light chain 4, axonemal-like | 8.27247E-42 | 2 |
| Transcript_AVA3_47460 | 196 | 20 | PREDICTED: dynein heavy chain 7, axonemal isoform X2 | 1.24682E-33 | 9 |
| Transcript_AVA1_28773 | 146 | 20 | sex-determining protein DMT | 2.53528E-22 | 0 |
| Contig_Tilapia_90_2942 | 766 | 20 | collagen, type I, alpha 2 precursor | 1.01398E-124 | 2 |
| Contig_Tilapia_90_9321 | 442 | 20 | PREDICTED: charged multivesicular body protein 2a-like | 1.11206E-51 | 2 |
| Transcript_AVA2_10563 | 138 | 20 | PREDICTED: 40S ribosomal protein S10-like | 2.60965E-22 | 9 |
| Transcript_AVA1_64597 | 135 | 0 | - | - | 0 |
| Contig_Tilapia_90_23367 | 207 | 0 | - | - | 0 |
| Transcript_AVA1_66083 | 148 | 0 | - | - | 0 |
| Transcript_AVA3_28576 | 490 | 0 | - | - | 0 |

^a^ Names of the sequences as they appear at [http://vmdiva-proto.ird.fr](http://vmdiva-proto.ird.fr/).

^b^ The number of BlastX hits was limited to 20.

^c^ According to the best blast hit.
